# Manganese(II) Oxidizing Bacteria as Whole-Cell Catalyst for β-Keto Ester Oxidation

**DOI:** 10.3390/ijms21051709

**Published:** 2020-03-02

**Authors:** Juan Guo, Huan Guo, Jin Liu, Fangrui Zhong, Yuzhou Wu

**Affiliations:** 1 Hubei Key Laboratory of Bioinorganic Chemistry and Materia Medica, School of Chemistry and Chemical Engineering, Huazhong University of Science and Technology, Wuhan 430074, China; guojuan0201@163.com (J.G.); d201980126@hust.edu.cn (H.G.); chemzfr@hust.edu.cn (F.Z.); 2 Max Planck Institute for Polymer Research, Ackermannweg 10, 55128 Mainz, Germany

**Keywords:** *Pseudomonas putida* MnB1, biogenic manganese oxides, abiotic manganese oxides, α-Hydroxy-β-keto esters, whole-cell biocatalysis

## Abstract

Manganese oxidizing bacteria can produce biogenic manganese oxides (BMO) on their cell surface and have been applied in the fields of agriculture, bioremediation, and drinking water treatment to remove toxic contaminants based on their remarkable chemical reactivity. Herein, we report for the first time the synthetic application of the manganese oxidizing bacteria, *Pseudomonas putida* MnB1 as a whole-cell biocatalyst for the effective oxidation of β-keto ester with excellent yield. Differing from known chemical protocols toward this transformation that generally necessitate the use of organic solvents, stoichiometric oxygenating agents and complex chemical catalysts, our strategy can accomplish it simply under aqueous and mild conditions with higher efficiency than that provided by chemical manganese oxides. Moreover, the live MnB1 bacteria are capable of continuous catalysis for this C-O bond forming reaction for several cycles and remain proliferating, highlighting the favorable merits of this novel protocol for sustainable chemistry and green synthesis.

## 1. Introduction

Developing sustainable biocatalytic processes for chemical synthesis has attracted considerable attention due to the ever-increasing environment concerns [1,2,3]. Conventional chemical production provides organic compounds that fulfil fundamental demands of modern society in pharmaceutical, agricultural, material and other fields, however, often at the expense of environment pollution and energy consumption. As such, biocatalysis provides a more favorable alternative considering its merits such as high catalytic activity and selectivity, mild reaction conditions (physiological pH and temperature), and environmental credentials (enzymes, organic solvent-free medium) [4,5,6]. In particular, whole-cell biocatalysis possesses unique advantages and extraordinary attractiveness. First, enzymes inside cells are to some extent in a protected environment and therefore often more stable than their isolated counterparts [7]. Besides, whole-cell biocatalysis integrates the benefits of enzyme cascades in a bacterial system and the fast proliferation of a living microbe, thus being more energy efficient, sustainable and easily recyclable [8]. However, the whole-cell catalytic reactions necessitate fast transportation of non-toxic substrates across the cell envelope to contact the enzymes, which essentially limits the substrate scope and reaction rate [9]. Therefore, novel strategies to utilize microorganisms for useful organic transformations are demanded to broaden the application of whole-cell biocatalysis in sustainable synthesis of fine chemicals.

**Figure 1:**
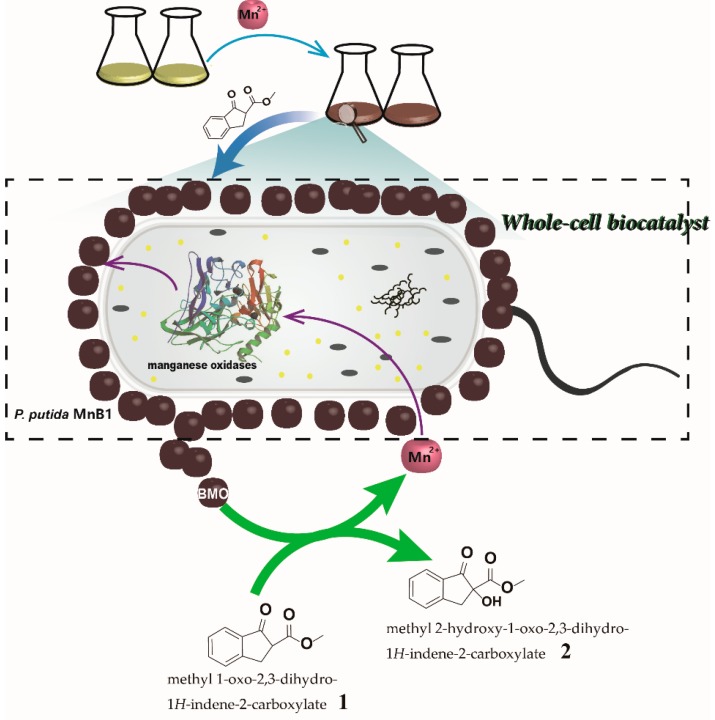
α-hydroxy-β-keto ester (**1**) by whole-cell biocatalysis based on biogenic manganese oxides (BMO).

Manganese dioxide (MnO_2_) is a classic oxidant in organic synthesis with broad substrate scope and high reaction selectivity, as seen in alcohol oxidation, aromatization, oxidative coupling, and thiol oxidation [10,11,12,13,14]. In nature, biogenic manganese oxides (BMO) produced by Mn(II) oxidizing bacteria is widely present in soil and sediment, which has been extensively studied as a chemical catalyst or oxidizing reagent to remove various organic pollutants [15,16,17]. Of note, the main content of BMO is MnO_2_, which was found to have even larger specific surface area and higher reactivity than chemically prepared equivalents [18,19]. BMO producing bacteria can be directly applied in the fields of agriculture, bioremediation, and drinking water treatment to remove toxic contaminants [20,21,22,23], exhibiting extraordinary advantages such as high efficiency, low cost and environmental safety. Moreover, since the BMO is produced on the surface of bacteria and even secretes to the environment, these microbes can catalyze reactions without requiring the cell uptake of substrates and thus might benefit the reaction kinetics. Despite remarkable advances in various fields, the use of Mn(II) oxidizing bacteria as a whole-cell catalyst for synthesizing fine chemicals has not been explored (Figure 1).

*Pseudomonas putida* MnB1, one of the most studied Mn(II) oxidizing bacteria, is ubiquitous in freshwater and soil, and can be cultivated even in complicated environments [20]. It would oxidize Mn(II) in liquid and solid media to Mn(IV) and accumulate BMO precipitates on the cell surface [24]. The robustness of *P. putida* MnB1 lays the groundwork for their prospective synthetic application as potential biocatalyst. To prove the concept of Mn(II) oxidizing bacteria whole-cell biocatalysis for organic synthesis, α-hydroxylation of β-keto ester (**1**) (methyl 1-oxo-2,3-dihydro-1H-indene-2-carboxylate) was selected as model reaction. This reaction provides the most straightforward access to the α-hydroxy-β-dicarbonyl, an intriguing moiety commonly found in various biologically active natural products, agrochemicals, and pharmaceuticals [25,26,27]. Notably, a number of chemical protocols are available to accomplish this oxidation to yield product **2** [28,29,30,31,32,33]. For instance, Lu et al. reported a Brønsted acid catalytic method with nitrosobenzene as the oxygen source [28], and Meng and co-workers documented a Zr(IV)/organic peroxide system [30]. In general, the use of organic solvents and stoichiometric oxygenating agents were necessitated in conjunction with complex chemical catalysts, thus strongly compromising reaction economy and environmental friendliness. Herein, the BMO-based MnB1 catalyzed α-hydroxylation of β-keto ester (**1**) can be successfully achieved in water with superior performance than that of chemically produced MnO_2_. Moreover, the live MnB1 bacteria can be recycled with ease and remain proliferating, thus they are capable of continuously catalyzing the conversion of substrates. Therefore, this is a sustainable whole-cell biocatalytic system for efficient oxidation reaction that holds marked advantages for industrial applications due to the high efficiency, low cost and potential for flow chemistry.

## 2. Results

### 2.1. Biogenic Manganese Oxides (BMO) Formation

The reaction was firstly tested with lyophilized powder of the BMO mineralized MnB1 bacteria (noted as dry m-MnB1). To prepare it, MnB1 was cultured in Lept medium containing 1 mM Mn^2+^ for mineralization and the formation of BMO was observed by the production of dark brown sediments after 35 h. The bacteria were continuously cultured for 5 d before harvesting by centrifugation and lyophilization. The Mn content in the dry m-MnB1 was determined to be 28.8% according to inductively coupled plasma optical emission spectrometer (ICP-OES) (Figure S1). This result is consistent with the respective literature data (18–30%) [18,19].

### 2.2. Reactivity Comparison of the Dry m-MnB1 with Chemical Manganese Oxides (CMO) toward the Oxidation of β-Keto Ester

#### 2.2.1. Solvent Effects on Oxidation Rate

**Figure 2:**
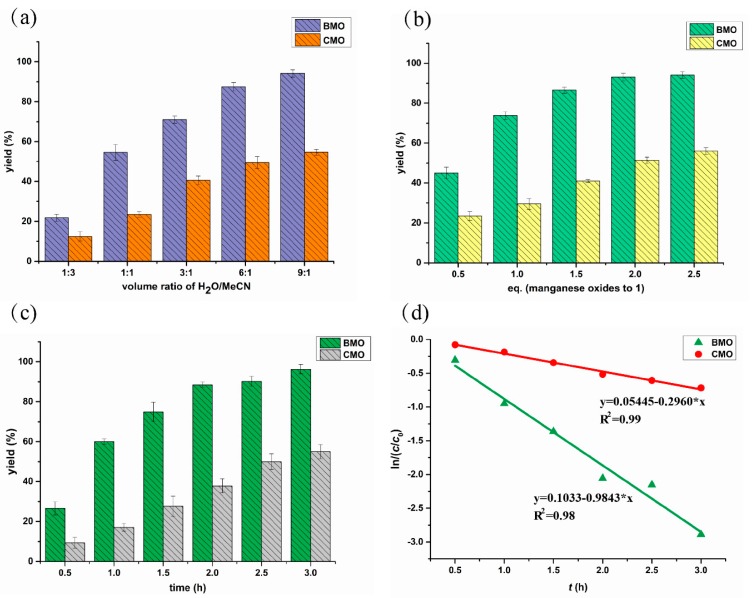
Comparing the synthesis of α-hydroxy-β-keto ester by the BMO (biogenic manganese oxides) from dry m-MnB1 and CMO (chemical manganese oxides): (**a**) Effects of reaction media consisting of H_2_O/MeCN with different ratios; (**b**) Effects of dosage of BMO and CMO in H_2_O/MeCN (9:1); (**c**) Correlation of yield of **2** with time; (**d**) Pseudo-first-order plots of α-hydroxylation of β-keto Ester by BMO and CMO in the first 3 h.

The dry m-MnB1 containing the BMO lyophilized together with MnB1 bacteria was mixed with β-keto ester (**1**) to test the oxidation reactivity. The performance of dry m-MnB1 was first compared with commercially available MnO_2_ powder (CMO) in different media consisting of H_2_O and acetonitrile (MeCN) under otherwise identical conditions. The Mn content was adjusted to be the same for both dry m-MnB1 and CMO. The oxidized product was characterized by ^1^H NMR spectroscopy (Figure S2). The yield of product **2** was determined by high performance liquid chromatography (HPLC) using comparably normalized standards (Figure S3). It was found that the mixed solvent system comprised of 90% H_2_O and 10% MeCN gave the highest yield of product 2 for both BMO and CMO (3 h, 94.1% and 54.7%, respectively) (Figure 2a). Increasing the organic proportion was detrimental. For instance, the respective yield dropped to 21.8% and 12.7% in MeCN/H_2_O (3:1). However, a small amount of MeCN was still necessary to dissolve the organic substrate. Nevertheless, the aqueous system adopted herein is much more favorable than known protocols exclusively using organic solvents.

Notably, in all tested solvent conditions, BMO in dry m-MnB1 revealed significantly higher reactivity than CMO, and almost 2-fold higher yields were attainable in any given medium (Figure 2a). These results proved that the dry m-MnB1 possesses superior reactivity and selectivity than conventional CMO.

#### 2.2.2. Influence of Dosage of Manganese Oxides on Reaction Rate

We subsequently explored the effects of dosage of manganese oxides on the oxidation of compound **1**. A mixture of H_2_O/MeCN (9:1) was employed as the reaction medium and the reaction time was set at 1 h. As shown in Figure 2b, the ratio of manganese oxides to substrate **1** was found to significantly impact the oxidation process. As the dosage of BMO from dry m-MnB1 was increased from 0.5 eq. to 1.0 eq., the yield of **2** raised from 42% to 72%. The outcome could be further improved to 91% with 2.0 eq of oxidant, and it became marginally higher with further addition of BMO. These results suggest 2.0 eq. of BMO is adequate. As the substrate **1** dissolved in the reaction medium was originally 5.0 mM, the factor limiting it from approaching full conversion might be the extremely low concentration at this point. Analogously, the same set of experiments were conducted with CMO. Although the conversion of substrate **1** was also evidently promoted along the addition of oxidant, the yield of **2** was constantly lower than BMO under identical conditions, for instance, as seen from 56% yield versus 93% yield with 2.5 eq. of manganese oxides. The results also back the notion that the BMO from dry m-MnB1 is more efficient than the CMO for this oxidation reaction.

#### 2.2.3. Reaction Kinetics

The kinetic parameters for both BMO and CMO mediated oxidation were studied (Figure 2c,d). It was found that α-hydroxylation of substrate **1** with both BMO from dry m-MnB1 and CMO in the first 3 h followed Pseudo-first order kinetics, which were expressed as in equation (1), with *C*: concentration of 1 (mmol/L) at time *t* (h), *C*_0_: the initial concentration of **1** (mmol/L), and *k*: the rate constant (h^−1^). By transforming Equation (1) into Equation (2),

(1) $$C=C_{0}\cdot e^{-k\cdot t}$$

(2) $$ln\left(\frac{c}{c_{0}}\right)=-k\cdot t$$

the rate constant can be calculated from the slope of the graph as depicted in Figure 2d. An average value for the rate constant *k* of 0.9843 ± 0.0215 h^−1^ was calculated with BMO from dry m-MnB1, while on the contrary, the rate constant of CMO was merely 0.296 ± 0.0286 h^−1^ (Table 1). It is clear that the oxidation activity of the BMO from dry m-MnB1 is higher than CMO.

### 2.3. Effects of β-Keto Ester 1 on Bacteria Growth

**Figure 3:**
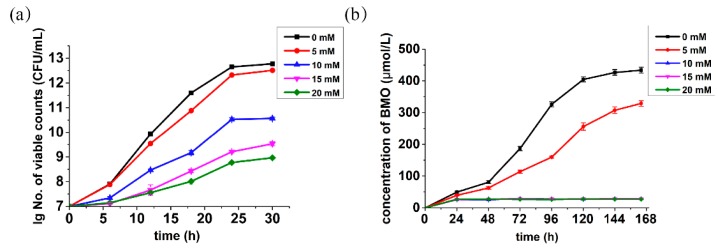
Effects of β-keto ester on the bacteria growth (**a**) measured by spread plate method and (**b**) the manganese mineralization; various colors refer to different concentrations of **1**; CFU: colony forming units.

In order to achieve the α-hydroxylation of **1** by directly using live MnB1 bacteria, we moved on to investigate the influence substrate **1** on the growth of *P.* *putida* MnB1 at different concentrations. As illustrated in Figure 3a and Figure S4, the MnB1 growth profile did not deviate notably from the standard strain incubation in the presence of 5.0 mM of substrate, while larger concentration (10.0 mM) exhibited obvious toxicity. Further increase of concentration to 15.0 and 20.0 mM both significantly suppressed the bacterial growth. Nevertheless, the bacteria were still proliferating even with 20.0 mM substrate, highlighting the robustness of MnB1.

### 2.4. Effects of β-Keto Ester 1 on Manganese Mineralization

The effects of various concentrations of **1** on the manganese mineralization by the model strain were also probed. However, the manganese mineralization of MnB1 was found to be much more sensitive to substrate **1**. A concentration of 5.0 mM has already shown a significant inhibition on mineralization process, which was found to be completely repressed at 10.0 mM or higher (Figure 3b). Since the generation of BMO by *P. putida* MnB1 was fundamentally accomplished via multicopper oxidase-catalyzed oxidation of Mn(II) into Mn(VI) in cell extracts within its active center with three Cu(II) sites [34,35,36], and the β-keto ester is known to chelate divalent metal ions, we speculate that compound **1** might potentially act as a multicopper oxidase inhibitor [37] to prohibit manganese oxide mineralization. Therefore, a maximum substrate concentration of 5.0 mM was selected for the following whole-cell biocatalytic oxidation of substrate **1**.

### 2.5. Continuous Live MnB1 Catalyzed Synthesis of α-Hydroxy-β-Keto Ester

**Figure 4:**
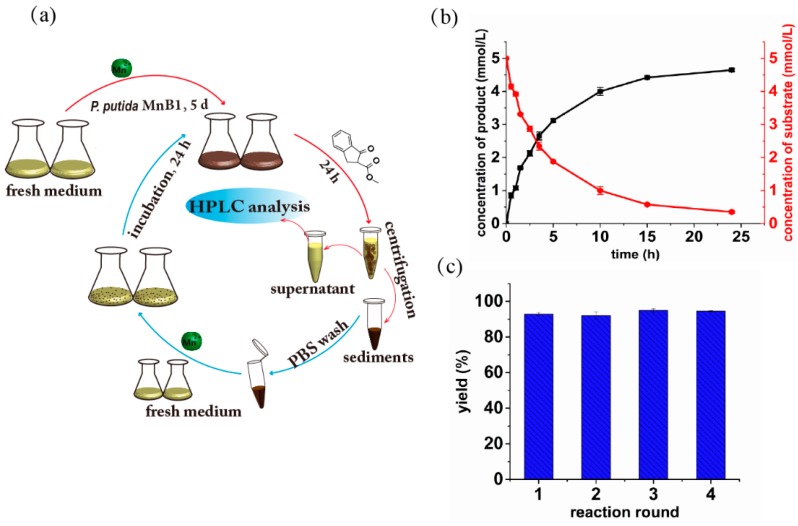
Synthesis of α-hydroxy-β-keto ester by the whole cells based on the biogenic manganese oxides: (**a**) Schematic diagram of the recycling experiments; (**b**) Profile of substrate consumption and product formation; (**c**) Yield of product **2** in recycling reaction.

As the whole-cell catalysts have been previously demonstrated as a continuous and repeated-batch reaction system [38], herein we explored such potential of live MnB1 for α-hydroxylation of **1**. The MnB1 bacteria were cultivated in Lept medium containing 1.0 mM MnCl_2_ until 0.45 mM BMO were formed according to leucoberbelin blue (LBB) test [39]. The substrate **1** was then directly added to the culture medium to reach the concentration of 5.0 mM. During the first round of synthesis, the reaction progress was monitored as shown in Figure 4a. The reaction rate was fast at the initial stage but slowed down with the BMO being consumed. The highest conversion could reach 92% after 24 h (Figure 4b). Afterwards, the MnB1 bacteria were recycled by centrifugation, and washed by PBS before re-culturing in a fresh medium. The same concentration of fresh substrate was added to the medium when the BMO concentration reached 0.45 mM again. The yield of product **2** was consistently determined after 24 h. Notably, in consecutive four cycles, virtually identical yield was attainable for each run, and all were over 90% (92%, 91%, 93% and 94% respectively, Figure 4c). These results clearly suggest that the MnB1-based whole-cells catalyst can be easily regenerated and recycled for continuous reactions, which is highly valuable for low cost and sustainable catalysis and flow chemistry.

## 3. Discussion

Manganese oxides are known to be generated via oxidation of Mn^2+^ through both abiotic and biotic channels in the environment. Related reports indicated that the abiotic manganese oxidation pathway is much slower than biological processes, which are performed by a large variety of bacteria and fungi [40,41]. Here, we used *P. putida* MnB1 as the model strain to generate the BMO. Notably, *P. putida* MnB1 is ubiquitous in the freshwater and soil, and its optimal growth temperature is 26~30 °C [20], which indicates that the strains are readily available and can be cultivated even in complicated environments. Thus, the robustness of *P. putida* MnB1 lays the groundwork for the prospective industrial application of BMO.

In recent years, BMO have received broad attention due to its marked redox reactivity, for instance, for the degradation of the organic pollutants, removal of heavy metal ions, etc. [42,43]. Although these processes involve various organic transformations, no rational synthesis of organic compounds with specific bond formation using BMO has been disclosed. In this study, we realized the synthesis of valuable α-hydroxy-β-keto ester **2** by utilizing BMO as an oxidant to promote the highly selective C-O bond formation event. Its superior performance than commercially available CMO highlights a strong capacity for synthetic oxidative reactions, and this is consistent with the previous studies on degradation applications [44]. In fact, oxidation of substrate **1** with BMO was a spot-to-spot process based on thin layer chromatography (TLC) analysis, and it was much faster and cleaner than that with CMO. Mechanistically, BMO produced by the *P. putida* MnB1 is known to contain larger inter-layer space and less structural Mn(III) than the CMO [45], thus providing larger specific surface areas [46]. We presume that these characteristics of BMO are beneficial to render defined interaction with substrates, and likely account for their better performance observed in our experiments.

Despite remarkable performance of isolated BMO for the oxidation of β-keto ester **1**, we were further motivated to develop an environmentally friendly and efficient catalytic system by using whole-cell bacteria as a novel type of catalyst. However, tremendous challenges need to be taken into consideration, such as the compatibility with reaction media, the toxicity of substrate to cells and others [47]. Results of solvent optimization (Figure 2a) guided us to take mixture of H_2_O and MeCN as a suitable reaction medium. However, toxicity experiments showed that higher concentration (10 mM) of substrate would drastically inhibit the mineralization function of bacteria (Figure 3b). Jung [35] and Francis [34] independently concluded that the generation of BMO by *P. putida* MnB1 was fundamentally accomplished by multicopper oxidase, which catalytically oxidizes Mn(II) into Mn(VI) in cell extracts within its active center with three Cu(II) sites. This activity would get inhibited by heating or by treatment with a protease. The β-keto ester is known to chelate divalent metal ions, an essential component in the active site that interacts with amino acid residues of the enzyme. Therefore, this type of substrate might potentially act as a protein inhibitor [37]. We estimated that the higher concentration of β-keto ester **1** suppressed the manganese oxidizing partially due to the toxicity of substrate to the bacteria growth (Figure 3a and Figure S4), and this was probably a result of the impact of substrate on the activity of the multicopper oxidase. Based on these findings, we chose 5 mM as an appropriate concentration of substrate to conduct the whole-cell biocatalysis. As the content of β-keto ester **1** was 5-fold to Mn(II), the obtained 92% yield of product **2** clearly indicates Mn is operative in a catalytic manner, i.e., the regeneration of BMO by *P. putida* MnB1. Although a similar notion has been offered in the degradation of organic pollutants by Ko et al. [18], however, the recyclability of the bacteria for continuous catalysis has never been demonstrated.

In conclusion, we have realized the synthesis of valuable α-hydroxy-β-keto ester (**2**) by utilizing BMO as an oxidant to promote the highly selective C-O bond formation. Its superior performance than commercially available CMO highlights a strong capacity for synthetic oxidative reactions. Consecutive repeated-batch synthesis with recovered bacteria by the whole-cell catalytic system was achieved with consistently high levels of yield recorded [38]. Collectively, the whole-cell catalytic *P. putida* MnB1 with biogenic manganese oxides is highly robust, even amenable to organic transformation with somewhat toxic substrates. As Mn(IV) is extensively utilized in oxidative organic reactions, we anticipate that this efficient biological system is promising for the benign synthesis of various bioactive substances as well as bulk fine chemicals to meet the increasing demand for sustainable chemistry.

## 4. Materials and Methods

### 4.1. Preparation of Freeze-Dried BMO

The bacterial strain *Pseudomonas* *putida* MnB1 [American Type Culture Collection (ATCC) no. 23483] was cultured in the Lept medium (0.5 g/L yeast extract (Ruji, Shanghai, China), 0.5 g/L Casamino Acids (Coolaber, Beijing, China), 5 mM D(+)-glucose (Macklin, Shanghai, China), 10 mM HEPES (*N*-2-hydroxyethylpiperazine-*N*’-2-ethanesulfonic acid, pH 7.5, GBCBIO, Guangzhou, China), 0.48 mM CaC1_2_ (Macklin), 0.83 mM MgSO_4_ (Macklin), 3.7 μM FeCl_3_ (Macklin), and 1 mL of trace element solution (10 mg/L CuSO_4_•5H_2_O, 44 mg/L ZnSO_4_•7H_2_O, 20 mg CoCl_2_•6H_2_O and 13 mg/L Na_2_MoO_4_•2H_2_O, (Macklin)) containing 1mM MnCl_2_ (Aladdin, Shanghai, China) at 30 °C and shaken at 150 rpm for 5 d [48]. The suspensions were centrifuged at 8000× *g* for 20 m and the supernatant was discarded, sediments of bacteria and BMO were diluted three times with deionized water by means of centrifugation (20 m at 8000× *g*), then the mixture of precipitates were collected for freeze-drying to obtain the dried BMO sample.

### 4.2. Quantification of Freeze-Dried BMO by Inductive Coupled Plasma Optical Emission Spectrometry (ICP-OES)

The 25 μg dry m-MnB1 was treated with 200 μL 90% HNO_3_ overnight and heated at 70 °C for 30 min, then was added H_2_O_2_ (30%, 200 μL). The resulting mixture was treated to evaporate at 200 °C for 1 h and then dissolved in deionized water to 4 mL solution to be measured by Inductive Coupled Plasma Optical Emission Spectrometry (ICP-OES 7000 Plus, ThermoFisher, Waltham, MA, USA) to quantify the amount of Mn^2+^. ICP-OES combined with a water cross-flow nebulizer and Ar was run as the carrier gas; auxiliary energy flow, coolant flow, and nebulizer flow were set as 0.7 L/min, 13.00 L/min, and 0.7 L/min, respectively. The analyses were calibrated by gravimetric standards with different concentrations (2 ppm, 4 ppm, 8 ppm, 16 and 32 ppm, respectively) that were measured before sample quantification.

### 4.3. Synthesis of α-Hydroxy-β-Keto Ester by the Dry m-MnB1 and CMO

A reaction mixture (400 μL, MeCN: H_2_O = 1:1) containing 50 mM β-keto ester **1**, 5.0 mg dry m-MnB1 or 1.75 mg CMO was shaken at 150 rpm, 30 °C for 3 h. A control experiment was conducted without manganese oxide under otherwise identical reaction conditions. All reaction mixtures were monitored by TLC (hexanes/ethyl acetate = 3:1). Then, the resulting samples were centrifuged at 8000× *g* for 5 m, supernatant was extracted with dichloromethane and evaporated under reduced pressure. The dried residues were purified by silica gel chromatography with hexanes/ethyl acetate (20:1–10:1) to get desired products for the NMR analysis. Finally, the purified samples were used as standards for the following HPLC detection.

#### 4.3.1. Optimization of the Reaction Medium

The reaction mixtures (400 μL, MeCN: H_2_O = 3:1, 1:1, 1:3, 1:6 or 1:9) containing 50 mM β-keto ester (1) (Preparation of this substrate followed a known protocol [49]), 5.0 mg dry m-MnB1 or 1.75 mg CMO (Aladdin) were shaken at 150 rpm, 30 °C for 3 h. The reaction samples were centrifuged at 8000× *g* for 5 m and supernatant was analyzed using HPLC to calculate the yield of oxidation product.

#### 4.3.2. Assays of Different Dosage of Manganese Oxides on the Oxidation Reaction

Substrate **1** (0.02 mmol) and dry m-MnB1/CMO (Chemical manganese oxides, Innochem, Beijing, China) (0.01, 0.02, 0.03, 0.04, or 0.05 mmol) were added to test tubes and dissolved in MeCN (40 μL) and H_2_O (360 μL). The reaction samples were stirred at 150 rpm, 30 °C for 1.5 h. Then, the reaction systems were centrifuged at 8000× *g* for 5 m and supernatants were analyzed using HPLC to obtain the yield of the oxidation product.

#### 4.3.3. Kinetic Measurements of Dry m-MnB1 and CMO

To further compare the reaction efficiency of dry m-MnB1 and CMO, kinetic parameters were detected. The reaction mixtures (**1** (0.02 mmol), dried-BMO/CMO (0.02 mmol), MeCN (40 μL) and H_2_O (360 μL) were stirred at 150 rpm, 30 °C, and the reaction progress was monitored by yield of the oxidation product.

### 4.4. Bioassays of Substrate β-Keto Ester Tolerance of Pseudomonas putida MnB1

The bacterial strain *Pseudomonas putida* MnB1 was precultured overnight in LB medium (10 g/L tryptone, 10 g/L NaCl, 5 g/L yeast extract) at 150 rpm, 30 °C and transferred into fresh Lept medium containing 1 mM MnCl_2_ and various concentrations of **1** (0 mM, 5 mM, 10 mM, 15 and 20 mM, respectively). The colony forming units (CFU) were quantified by using the standard spread plate method [50] every 5 h until the generation of BMO. Simultaneously, the OD_600 nm_ level of bacteria was performed with microplate reader (3020-675, ThermoFisher).

### 4.5. Investigation of the Effects of Substrate on Manganese Mineralization

The oxidized Mn content generated in the suspension that was described in the Section 4.1 was measured at the indicated time points by using leucoberbelin blue (LBB, Sigma-Aldrich, St. Louis, MO, USA) method as illustrated by Krumbein [39]. With negligible modifications, bacteria culture (10 μL) was added to 50 μL LBB. After 15 m in the dark at room temperature, deposits were removed by centrifugation. KMnO_4_ was used as the standard to confirm the absorbance at 620 nm of supernatant.

### 4.6. Continuous Biocatalytic Experiments by Whole Cells Based on BMO

The bacterial strain *Pseudomonas putida* MnB1 was cultured in the Lept medium containing 1 mM MnCl_2_ at 150 rpm, 30 °C for 5 d to generate the BMO. The concentration of BMO was quantified by LBB method before adding the 5 mM substrate **1** to the medium. The mixture was then continued to shake at 150 rpm, 30 °C to get target product and the reaction progress was monitored by HPLC analysis. After the first-round reaction, the bacteria with BMO were collected by centrifugation and diluted three times with deionized water. The precipitates were added to fresh Lept medium containing 1 mM MnCl_2_ to allow the continuous mineralization of manganese at 30 °C and 150 rpm. The mixture was used for the second-round reactions under comparable conditions when the concentration of BMO was similar with the first-round initial content. Four consecutive rounds of biocatalysis by whole-cell were conducted and yield of product in each round was measured. All procedures were performed under rigorous aseptic conditions.

### 4.7. Methods and Conditions of HPLC Analysis

For HPLC detection, all of the collected supernatant samples were filtered over a 0.22 μm filter (Millipore, Billerica, MA, USA) and analyzed by HPLC system containing a LC-UV 100 absorbance detector (Wufeng, Shanghai, China). The compounds were separated on a reverse phase HPLC C18 column (C18 250 × 4.6 mm, 5 μm, Shodex, Tokyo, Japan) at a constant flow rate of 1.0 mL/min and analysed by UV/Vis detection at 254 nm. Solvent A was deionized water containing 5% trifluoroacetic acid (TFA, Aladdin) and solvent B was acetonitrile containing 5% TFA. A gradient from 0% to 70% solvent B was applied from 0 to 5 min, the solvent B decreased 70% to 55% from 5 to 20 m, then dropped to 5% during 5 m. All analyses were calibrated by the absorption of substrate and product standards with different concentrations.

### 4.8. Statistical Analysis

All data were presented as means ± standard deviation of three replicates. Statistical analysis was completed using SPSS 16.0 (Statistical Package for the Social Sciences, IBM Corp., Armonk, NY, USA).

## Figures and Tables

**Table 1:** Kinetic parameters of manganese oxides.

Data	Dried BMO ^1^	CMO ^2^
Rate constant (*k*, h^−1^)	0.9843 ± 0.0215	0.296 ± 0.0286
R^2^	0.97~0.98	0.98~0.99

^1^ BMO: biogenic manganese oxides; ^2^ CMO: chemical manganese oxides.

## Supplementary Materials

Supplementary materials can be found at https://www.mdpi.com/1422-0067/21/5/1709/s1.

## Abbreviations

BMO	Biogenic manganese oxide
CMO	Chemical manganese oxide
MeCN	Acetonitrile
CFU	Colony forming unit
ICP-OES	Inductive coupled plasma optical emission spectrometry
TFA	Trifluoroacetic acid
