# Supplementary material for: Manganese(II) Oxidizing Bacteria as Whole-Cell Catalyst for β-Keto Ester Oxidation
Source: Int J Mol Sci. 2020 Mar 2;21(5):1709. doi: 10.3390/ijms21051709 (PMC7084315; doi:10.3390/ijms21051709)
Supplement: Supplementary file 1 [file ijms-21-01709-s001.pdf]

Article

Manganese(II) Oxidizing Bacteria as Whole-Cell Catalyst for β-Keto Ester Oxidation

Juan Guo [1], Huan Guo [1], Jin Liu [1,*], Fangrui Zhong [1] and Yuzhou Wu [1,2,*]

[1] Hubei Key Laboratory of Bioinorganic Chemistry and Materia Medica, School of Chemistry and Chemical Engineering, Huazhong University of Science and Technology, Wuhan 430074, China; guojuan0201@163.com (J.G.); d201980126@hust.edu.cn (H.G.); chemzfr@hust.edu.cn (F.Z.)

[2] Max Planck Institute for Polymer Research, Ackermannweg 10, 55128 Mainz, Germany

* Correspondence: liujinlj1987@163.com (J.L.); wuyuzhou@hust.edu.cn (Y.W.)

Received: 14 February 2020; Accepted: 24 February 2020; Published: date

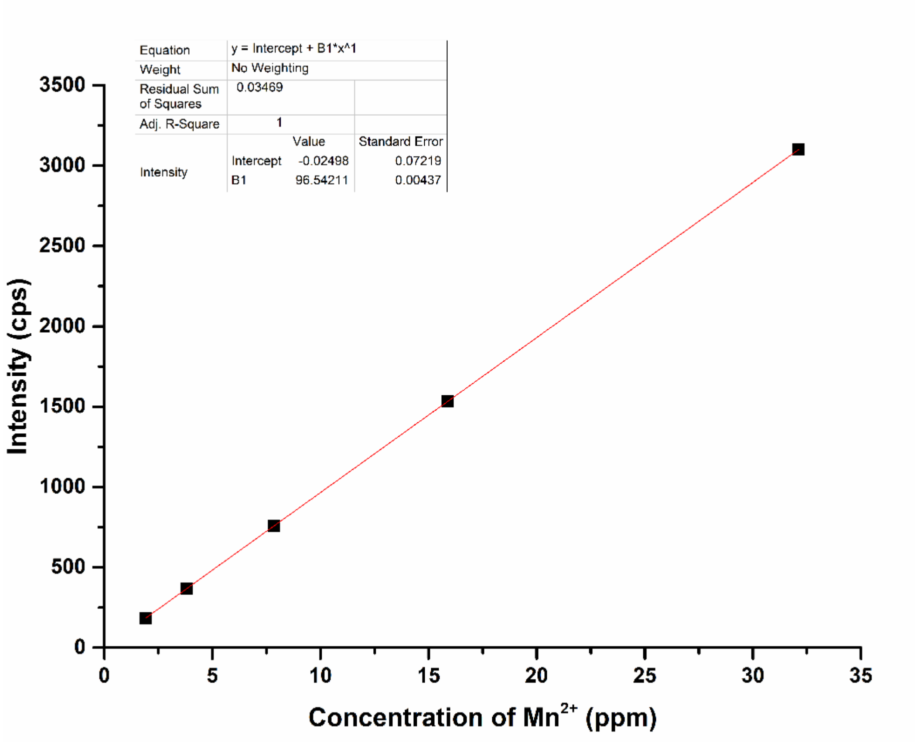

Figure S1. Standard curve of Mn^{2+}quantification by Inductive Coupled Plasma Optical Emission Spectrometry (ICP-OES); y=96.54211x - 0.02498, R^2=1.00000. (The actual concentration of Mn ions was 7.199 ppm in the 25 ppm dry m-MnB1, therefore, the content of Mn in the dry m-MnB1 was calculated as the following: 7.199/25=28.8%).

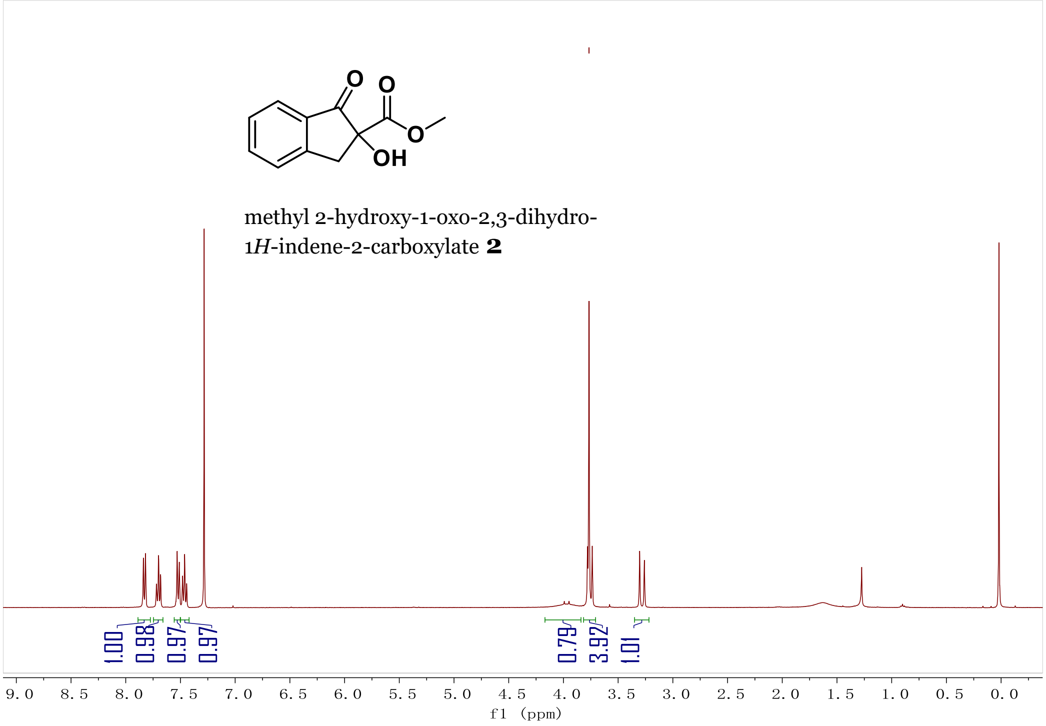

Figure S2. ^{1}H NMR of ◎-hydroxy-◎-keto ester **2**. ^{1}H NMR (400 MHz, Chloroform-d) δ 7.83 (d, *J* = 7.8 Hz, 1H), 7.70 (td, *J* = 7.5, 1.3 Hz, 1H), 7.52 (dt, *J* = 7.8, 1.0 Hz, 1H), 7.50 – 7.42 (m, 1H), 3.95 (s, 1H), 3.77 (m, 4H), 3.28 (d, *J* = 17.2 Hz, 1H).

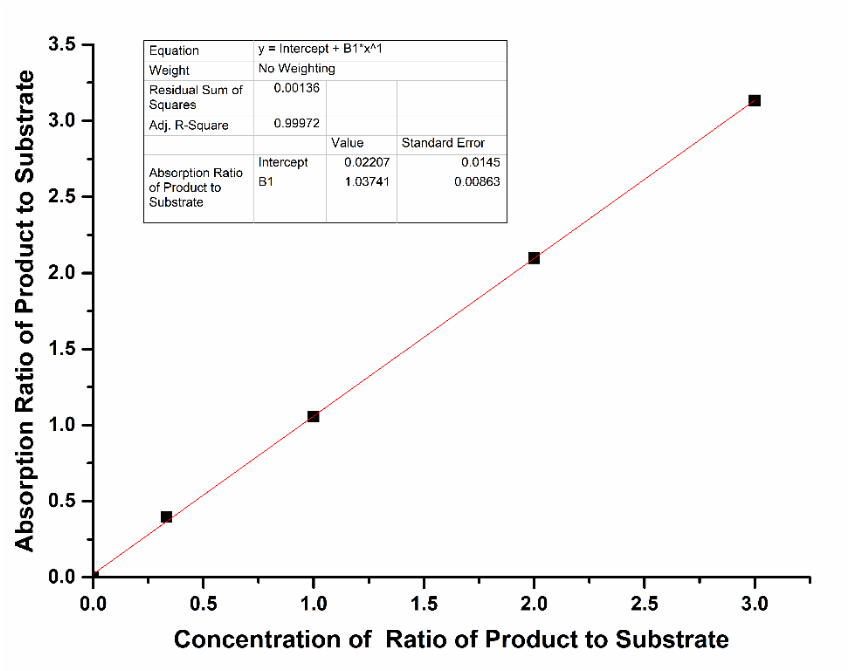

Figure S3. Standard curve of concentration of product to substrate which were measured by HPLC; y=1.03741x +0.02207, R^2=0.99972.

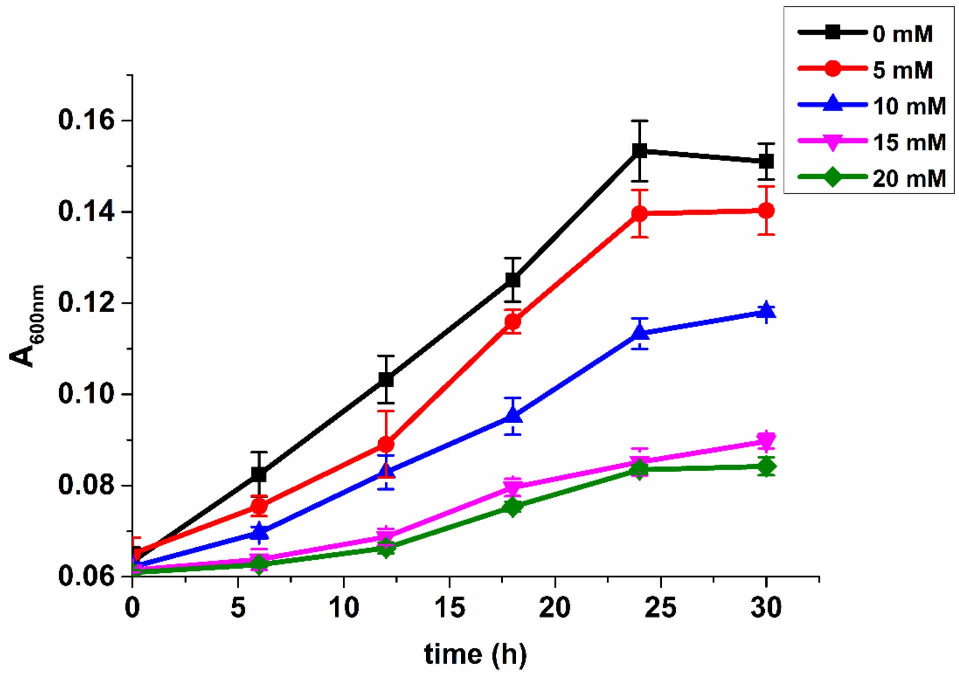

Figure S4. Effects of β-keto ester on the bacteria growth characterized by the OD620nm.
